# *GHRL* gene-based genotyping of ovine and caprine breeds reveals highly polymorphic intronic sequences in Awassi sheep with several RNA motifs

**DOI:** 10.1186/s43141-019-0004-5

**Published:** 2019-09-23

**Authors:** Mohammed Baqur S. Al-Shuhaib, Tahreer M. Al-Thuwaini, Israa A. Fadhil, Thamer R. S. Aljubouri

**Affiliations:** Department of Animal Production, College of Agriculture, Al-Qasim Green University, 8-Al-Qasim, Hillah, Babil 51001 Iraq

**Keywords:** Ghrelin, Goats, Sheep, SSCP, Genotyping

## Abstract

**Background:**

The current study was conducted to identify the genetic polymorphism of ghrelin (*GHRL*) gene of sheep and goats, as well as to determine whether these polymorphisms were associated with the evolutionary genetic differences in the involved species. This study was performed on 233 sheep and 91 goats. Two genetic loci of 113 bp and 262 bp partially spanning over exon 2/intron 2 and intron 4/exon 5 of *GHRL* gene respectively were amplified and genotyped using polymerase chain reaction–single-strand conformation polymorphism (PCR-SSCP) and DNA sequencing methods.

**Results:**

The SSCP banding pattern of 262-bp locus indicated the presence of four diplotypes (BC, BB, AC, and AB) in Awassi sheep, three diplotypes (BC, BB, and AB) in Karadi sheep, and only two diplotypes (BC and BB) in all goats’ samples. The current study detected several novel SNPs in the ovine–caprine populations as well as two SNPs that are observed only in sheep, including intron4:119 C>A and intron4:123 T>G. The phylogenetic analysis revealed that the observed diplotypes resided within ovine sequences and were closely related to caprine counterparts. Computational analyses indicated the presence of various intronic RNA motifs. However, all these motifs were gathered in Awassi breed.

**Conclusion:**

It is stated that the intron 4 is highly diverse amongst goats and sheep as well as within sheep with a particular emphasis on Awassi. This genetic peculiarity may in turn suggest a high polymorphic pattern of this breed in comparison with other related counterparts.

## Background

Ghrelin plays important roles in maintaining growth hormone release and energy homeostasis in vertebrates [1]. Ovine ghrelin is made up of 27 amino acids [2], which are encoded by *GHRL* gene that is located on the chromosome 19 and composed of 5 exons and 4 introns (NC_019476.2), while in goats, it is positioned in chromosome 22 and composed of only 4 exons and 3 introns (NC_030829.1). The *GHRL* gene polymorphisms have been associated with milk fat and protein synthesis in water buffaloes [3]; enhanced food intake, growth, and body conformation in cattle [4]; several growth traits in sheep [5]; and obesity in human [6]. Mutations in the *GHRL* gene could potentially cause a defective or inactive ghrelin hormone and alter growth hormone secretion and energy homeostasis [7]. In this regard, it can be emphasized that detection of the effect of *GHRL* genotype on the economically important phenotypic traits that it controls can only be possible through the identification of mutations that existed at the genome. On the other hand, it has been reported that the feeding regimes are significantly different amongst sheep and goats [8]. These variable regimes are maybe the consequences of the *GHRL* genetic variation status amongst them. Mutations can not only explore the gateway for searching molecular markers but also enable to break out the evolutionary relationship amongst animals of various species [9]. Keeping these facts in mind, the present study was undertaken to unravel the pattern of genomic diversity of both ovine and caprine *GHRL* gene through utilizing the SSCP technique that is characterized with its ability to identify the unknown mutations [10]. Besides, due to the lack of comparative knowledge about the genetic characterization and nucleotide sequence variations of *GHRL* gene amongst sheep and goat breeds, this study provides the first report on the detailed molecular characterization of the *GHRL* gene polymorphism in these highly worthwhile populations.

## Methods

### Blood sampling and DNA extraction

A total of randomly selected 324 animals that are maintained at different grazing herds in the middle Euphrates region of Iraq were included in this study. Regarding sheep (*Ovis aries*), both Awassi and Karadi breeds were included in the study. The studied native breeds of Awassi and Karadi constituted about 58% and 20% respectively of the national Iraqi sheep, which are characterized with their fat tail and carpet wool production and have some potential to produce milk [11]. Awassi and Karadi breeds are characterized by their ability to survive and reproduce under the condition of drought and extreme climate fluctuations [12]. Regarding goats (*Capra hircus*), both Shami and Native breeds were included in the present study. Blood samples were collected from 157 Awassi sheep, 76 Karadi sheep, 48 Shami goats, and 43 Iraqi Native goats. Genomic DNA was isolated using a salting out method [13]. The extracted genomic DNA was evaluated by 1% agarose gel electrophoresis in 1× TAE (40 mM Tris-acetate, 2 mM EDTA; pH 8.3) and quantified using a Nanodrop spectrophotometer (BioDrop μLITE; Biodrop, UK).

### PCR primer sequences

Two pairs of specific PCR primers were utilized in this study. The first PCR pair, including the forward primer 5′-CCCTGCTCTGGATGGACTTGGC-3′ and the reverse primer 5′-GGCTTTGGGCATTTAGGACGC-3′, was made of 113 bp [5]. This specific fragment partially covered the coding sequences (CDS) of exon 2 and its intronic downstream region of the ovine *GHRL* sequences (NC_019476.2), whereas the second PCR pair, including the forward primer 5′-GCCAAACTGGATGGCAACAG-3′ and the reverse primer 5′-AACAGACAGGTGGTTGGTCC-3′, that was made of 262 bp was designed in the present study using the NCBI primer BLAST software (https://www.ncbi.nlm.nih.gov/tools/primer-blast). This 262-bp *GHRL*-specific fragment partially covered intron 4, all the CDS of exon 5, and a partial downstream region of exon 5 of the same ovine *GHRL* sequences. The referred PCR amplicons were used to amplify both ovine and caprine DNA sequences.

### PCR

The PCR reaction was performed using the AccuPower PCR PreMix (Bioneer, South Korea). Each 20 μl of PCR premix contained 250 μM of dNTPs, 10 mM of Tris-HCl (pH 9.0), 30 mM of KCl, 1 U of Top DNA polymerase, and 1.5 mM of MgCl 2. The PCR reaction mixture was completed with 10 pmol of each primer and 30–50 ng of genomic DNA. The optimum annealing temperatures were determined empirically in our extracted genomic DNA template using a gradient PCR thermocycler (ver. Mastercycler-nexus; Eppendorf). The PCR program was as follows: initial denaturation at 94 °C for 5 min, followed by 30 cycles of denaturation at 94 °C for 30 s, annealing at 62.0 °C for 30 s, elongation at 72 °C for 30 s, and a final extension at 72 °C for 5 min. After performing the PCR thermocycling, the PCR products were verified by electrophoresis on a 1.5% (w/v) agarose gel in 1× TBE buffer (2 mM of EDTA, 89 mM of Tris-Borate, pH 8.3). All SSCP non-suitable PCR amplicon bands were eliminated.

### SSCP

SSCP experiments were performed according to Al-Shuhaib et al. [14] protocol with some modifications. Several parameters were optimized to avoid false positive results as well as to enhance sensitivity. Briefly, 2.5 μl of each amplification product was mixed with an equal volume of SSCP denaturing loading buffer (95% formamide, 0.05% xylene cyanol, 20 mM EDTA pH 8, and 0.05% bromophenol blue). The PCR amplicons were heat-treated at 95 °C for 10 min and chilled on ice for at least 5 min. Then, PCR amplicons were separated in a vertical mini-wide gel format, gel size (216 × 110 mm) with gel thickness 1.0 mm (model JY-CZ-B, Junyi-Dongfang Electrophoresis Equipment, China). Denatured PCR products were loaded into the wells of 8% acrylamide/bisacrylamide (37.5:1), containing 7% glycerol, and 1× TBE buffer. The gel was run at constant conditions (250 V/125 mA/210 min for both PCR amplicons) at room temperature. Gels were stained by silver nitrate [15] and photographed.

### DNA sequencing and sequencing analysis

Each of the varying patterns of the SSCP samples for polymorphic fragments was purified and sequenced from both directions (Macrogen Inc. Geumchen, Seoul, South Korea). Only evident chromatographs obtained from ABI sequence files were further analyzed, ensuring that the annotation and variations are not because of the PCR or sequencing artifacts. The sheep reference sequences of 262-bp locus were retrieved from NCBI websites (GenBank acc. no. NC_019476.2). Then, the sequenced diplotypes were edited, aligned, and compared with their referring sequences using the BioEdit Software, version 7.1 (DNASTAR; Madison, USA). The representative sequences of BC, AC, BB, and AB diplotypes were deposited into the NCBI GenBank database with the accession numbers MG387135–MG387138 respectively.

### Bio-computational analysis

The observed variations were analyzed by the FSPLICE, which is a web server that can provide the opportunity to search for both donor and acceptor splicing sites and to define their thresholds (http://www.softberry.com). The intronic regulatory motifs for referring sequences as well as the observed diplotypes were analyzed using RegRNA (a regulatory RNA motifs and elements finder) web server (http://regrna.mbc.nctu.edu.tw/html/prediction.html).

### Statistical analysis

The bio-statistical genetic diversity calculations were performed for the polymorphic 262-bp genetic fragment to estimate haplotype and diplotype frequencies, as well as Nei’s heterozygosity. These criteria were calculated with PopGene32 software, version 1.31 [16]. A *χ*^2^ test was determined to verify possible deviations from Hardy–Weinberg equilibrium (HW) expectations for the distribution of diplotypes. Average heterozygosity was employed to estimate genetic diversity within the population.

### Phylogenetic analysis

A comprehensive phylogenetic tree was constructed, in which the observed *GHRL* variants were compared with each other and with their *GHRL*-based DNA sequences of other livestock animals using NCBI-blastn server [17]. An inclusive tree was made using the neighbor joining tree option of Clustal Omega server (https://www.ebi.ac.uk/Tools/msa/clustalo/). Subsequently, a radial tree layout was made and visualized by Figtree software (http://tree.bio.ed.ac.uk/software/figtree/). The accession numbers of the tree-involved organisms were annotated and colored appropriately.

## Results

### SSCP banding patterns

**Fig. 1:**
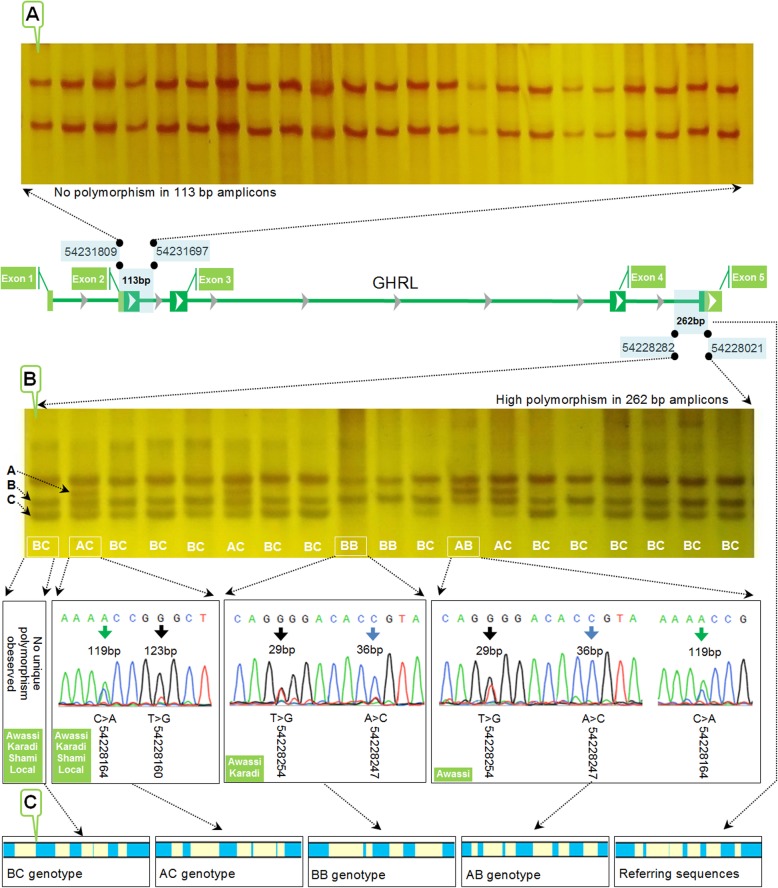
PCR-SSCP sequencing patterns of *GHRL* gene in Awassi, Karadi, Shami, and Iraqi Native breeds. **a** The monomorphic nature of the 113 bp spanning over exon2/intron 2. **b** The polymorphic nature of 262 bp spanning over intron 4/exon 5. **c** The in silico consequences of several RNA motifs predicted in the referring sequences as well as each observed diplotype of the 262-bp fragment

SSCP results showed the absence of any noticeable polymorphism in 113-bp fragment in all studied animals. Only two SSCP single-stranded DNA bands were revealed without any polymorphism in all 324 genomic DNA samples (Fig. 1a). In contrary to the 113-bp fragment, the 262-bp fragment showed four distinctly observed SSCP diplotypes, including BC, BB, AC, and AB, with three haplotypes, including A, B, and C. The SSCP results shown that the observed haplotypes were shared for both ovine and caprine fragments, while the distribution of these haplotypes was found to be different. The present study revealed two different SSCP diplotype distributions between sheep and goats. All four diplotypes were observed in Awassi sheep, including BC, BB, AC, and AB. In Karadi sheep, only three diplotypes were observed, including BC, BB, and AC with the absence of AB diplotype, whereas both BB and AB diplotypes were entirely absent in goats (Fig. 1).

### Sequencing interpretation

Sequencing results confirmed the observed four electrophoretic SSCP patterns as several unique SNPs were detected amongst three resolved SSCP banding patterns, including BB, AC, and AB. Sequencing of AC diplotype revealed that it was characterized by the presence of intron4:123 T>G variant, whereas intron4:119 C>A variant was found to be shared with AB diplotype. The latter diplotype also had two other variations that were shared in turn with BB diplotype, namely intron4:29 T>G and intron4:36 A>C. In contrary to other SSCP resolved banding patterns, sequencing experiments shown that the most abundant BC diplotype had no unique variation(s). However, six in common variations were observed in the intron 4 of all identified diplotypes including g.7 C>A, g.61 C>T, g.71 C>G, g.93 C>T, g.129 C>T, and g.149 C>T (Table 1). The novelty of all observed variations was confirmed by reviewing the corresponding *GHRL* referring sequences using the ensemble genome browser 95 (https://asia.ensembl.org/index.html).

**Table 1:** List of all single nucleotide polymorphisms observed in the SSCP diplotyped *GHRL* 262-bp amplicons in both sheep and goats

No.	Position*	BC diplotype	AC diplotype	BB diplotype	AB diplotype	SNP novelty	SNP summary
1	7	C>A	C>A	C>A	C>A	Novel	Intron4:g.7 C>A
2	29	–	–	T>G	T>G	Novel	Intron4:29 T>G
3	36	–	–	A>C	A>C	Novel	Intron4:36 A>C
4	61	C>T	C>T	C>T	C>T	Novel	Intron4:61 C>T
5	71	C>G	C>G	C>G	C>G	Novel	Intron4:71 C>G
6	93	C>T	C>T	C>T	C>T	Novel	Intron4:93 C>T
7	119	–	C>A	–	C>A	Novel	Intron4:119 C>A
8	123	–	T>G	–	–	Novel	Intron4:123 T>G
9	129	C>T	C>T	C>T	C>T	Novel	Intron4:129 C>T
10	149	C>T	C>T	C>T	C>T	Novel	Intron4:149 C>T

*The mentioned numbers refer to positions of the observed variants in PCR amplicons as well as in the submitted accession numbers (MG387135–MG387138) of the observed *GHRL*-based diplotypes. The observed variants were named following the nomenclature rules described in varnomen.hgvs.org/

### Bio-computational prediction

The FSPLICE prediction tool identified a noticeable alteration in the first position of the acceptor splicing site (1P) in all SSCP banding patterns. Various numbers in the threshold values for BC, AB, and BB diplotypes in comparison with the referring sequences were observed for the 1P, whereas this 1P position was entirely absent in AC diplotype (Table 2).

**Table 2:** Diplotype distribution and haplotype frequencies at the 262-bp *GHRL* locus in the Awassi and Karadi sheep and Shami and Iraqi Native goat breeds. The number of significant (*P* < 0.05) linkage disequilibria (LD) = 0. All chi-square tests have one degree of freedom

	Sheep		Goats	
	Awassi	Karadi	Shami	Native
BC diplotype (*n*)	94	41	36	39
BB diplotype (*n*)	34	30	–	–
AC diplotype (*n*)	9	5	12	4
AB diplotype (*n*)	20	–	–	–
Total	157	76	48	43
Haplotype A freq.	0.0924	0.0333	0.1277	0.0476
Haplotype B freq.	0.5796	0.6667	0.3723	0.4524
Haplotype C freq.	0.3280	0.3000	0.5000	0.5000
Total	1	1	1	1
Chi-square	44.71	21.62	46.00	41.00
Obs-Het	0.7834	0.6000	1.0000	1.0000
Exp-Het	0.5497	0.4676	0.6015	0.5496
Ave Het	0.5497	0.4644	0.5951	0.5431

To add another layer of confirmation, the regulatory intronic RNA motifs of all diplotypes were analyzed using RegRNA. RegRNA is an integrated in silico web server for identifying the homologs of regulatory RNA motifs and elements to determine their roles in the transcriptional and post-transcriptional regulation of gene expression [18]. We indicated the presence of variable intronic RNA regulatory motifs in the intronic sequences of all observed diplotypes (Fig. 1c).

### Statistical analysis

The most abundant SSCP diplotype was BC, which was observed in all studied population with a total frequency of 0.65 (*n* = 210). The highly dominant BC diplotype was followed by BB, which was found only in Awassi and Karadi sheep in a total frequency of 0.2 (*n* = 64). Then, AC diplotype, which was observed in all studied population, was recognized with only a small total frequency of 0.09 (*n* = 30), whereas it was found that AB diplotype that occupied the smallest total frequency of 0.06 (*n* = 20) was available only in Awassi sheep. The analysis of the *GHRL* 262-bp locus showed a prevalence of B haplotype, which is followed by C haplotype, while A haplotype was identified at a very low frequency in sheep in comparison to goats. The details of the diplotype distribution and haplotypes’ frequencies at the *GHRL* 262-bp locus are reported in Table 3.

**Table 3:** FSPLICE prediction of the donor and acceptor splice of both referring sequences and their diplotypes of *GHRL* 262-bp fragment for both sheep and goats

Diplotype	Acceptor(AG) sites, 1P: (134 W:) Threshold 4.175 (90%)	Acceptor(AG) sites, 2P: (213 W:) Threshold 4.175 (90%)	Donor(GT) sites, 1 P: (78 W:) Threshold 6.099 (90%)
Ref.	5.58 Seq: tctgcAGtggaa	8.47 Seq: atttcAGaaacc	6.10 Seq: gcactGTaaggc
AB	5.33 Seq: tttgcAGtggaa	8.47 Seq:atttcAGaaacc	6.10 Seq: gcactGTaaggc
BB	6.33 Seq: tttgcAGtggaa	8.47 Seq:atttcAGaaacc	6.10 Seq: gcactGTaaggc
AC	Not found	8.47 Seq: atttcAGaaacc	6.10 Seq: gcactGTaaggc
BC	6.33 Seq: tttgcAGtggaa	8.47 Seq:atttcAGaaacc	6.10 Seq: gcactGTaaggc

*Acceptor(AG) sites* the type of splicing sites, *P:* position of splicing site, *W:* weight of site. Threshold 4.175 (90%) means that for the current threshold value (4.175) 90% of true splicing sites are being classified as true

The *GHRL* locus was tested for the Hardy–Weinberg equilibrium and characterized by observed (obs-Het) and expected (exp-Het) heterozygosity in all included four populations. All studied populations did not follow the Hardy–Weinberg equilibrium at the *GHRL* locus and showed medium heterozygosity as obs-Het equals 0.7834 in Awassi, 0.6 in Karadi, 1 in Shami, and 1 in Native and exp-Het equals 0.5497 in Awassi, 0.4676 in Karadi, 0.6015 in Shami, and 0.5496 in Native breeds respectively. The values of the obs-Het for *GHRL* 262-bp diplotypes were higher than their expected values. This refers to the high level of genetic variability in the studied populations.

### Phylogenetic analysis

**Fig. 2:**
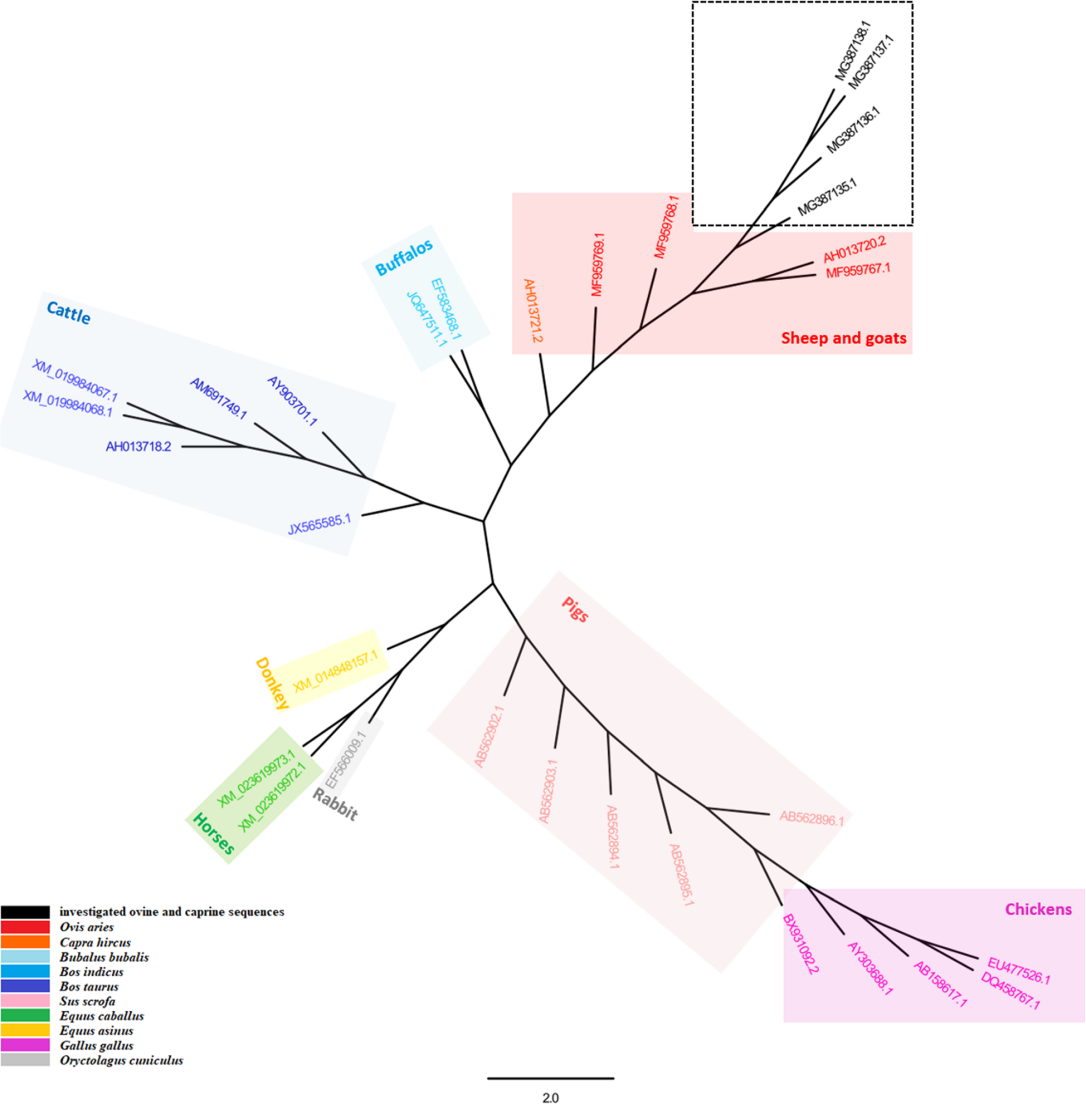
A *GHRL* gene-based phylogenetic tree of the observed 262-bp SSCP banding patterns of Awassi and Karadi sheep and Shami and Iraqi Native goats with other livestocks. Both query variants were colored and highlighted in black, while other livestocks were colored appropriately. All the mentioned numbers referred to GenBank accession number of each reference species. The number “2.0” at the bottom of the tree referred to the degree of scale range amongst the tree-categorized species

The phylogenetic analysis of the observed four diplotypes was conducted to explore the molecular taxonomic variations of *GHRL* variants. The phylogenetic tree provided a more detailed view of the specific taxonomic affinities shared by the investigated ovine–caprine sequences, reflecting their close evolutionary relationships. The total number of the aligned nucleic acid sequences, in addition to detected diplotypes, was 31, comprising the main livestock animals. The constructed phylogenetic tree revealed that all four *GHRL* diplotypes encompassed extremely close phylogenetic species positions (Fig. 2). These positions were positioned within ovine sequences, while caprine sequences occupied the nearest position in relation to their ovine counterparts. Unfortunately, the deposited caprine sequences were only one (AH0137212) and there were no other *GHRL*-based accession numbers regarding these sequences in caprine breeds, which may reduce the ability to exactly discriminate the observed diplotypes with the caprine species. However, the present phylogenetic tree demonstrated the phylogenetic positions and the close interactions between the observed diplotypes and with ovine and caprine sequences respectively.

## Discussion

Recently, genetic polymorphisms at candidate genes affecting economic traits have stimulated research interest because they are considered as an inevitable guide to genetic selection and to mark evolutionary relationships in different livestock breeds [19]. The use of DNA markers to account for genetic variation in metabolism, growth, and appetite controlling genes may provide a tool to assist geneticists in determining their exact role in the inter-species and intra-species relationship though focusing on their variations. The *GHRL* gene may represent an ideal candidate for this purpose.

Though we have examined two exonic regions spanning over exon 2 and exon 5 through 113 bp and 262 bp respectively, no missense mutations were observed and the structure of the protein does not vary between species. Meanwhile, the only high polymorphic region was found to be restricted on the fourth intron. In consonance with our findings, it was shown that all the observed *GHRL* SNPs were located in intronic or in the non-coding regions as in the water buffaloes [3], cattle [20], and goats [21]. However, this study reported the highly intronic variations of the *GHRL* 262-bp fragment and the lack of any polymorphism in the *GHRL* 113-bp fragment across the ovine and caprine species. Our *GHRL* 113-bp results were in agreement with earlier studies that have also reported the monomorphic nature of the same investigated locus in all examined ovine populations [5, 22]. Conversely, the highly polymorphic nature of the *GHRL* gene was successfully highlighted in the present study by designing the 262-bp fragment. The genotyping of the latter fragment has shown that Awassi breed was the most polymorphic one amongst the others since all four SSCP banding patterns were available in Awassi sheep. This observation could be attributed to the high nucleotide diversity ratios that accumulated in Awassi breed around the world [23]. Karadi breed was less polymorphic as being shown only three SSCP banding patterns. In contrary to ovine species, all studied caprine species were shown only two diplotypes irrespective of their breed. Our finding suggests that this deficiency may be due to sheep–goat intronic differences that have been unmasked by utilizing this 262-bp *GHRL* fragment. As the reported level of *GHRL* intron 4 variation is not similar in different species, it is rather acceptable to infer that the intronic *GHRL* gene expression pattern varies from species to species. Our findings of variable alternative patterns of the intronic RNA motifs and splicing of *GHRL* 262-bp locus may also suggest variable roles of these alternations in the transcriptional modifications [18]. It was shown that the variations of the *GHRL* gene affected gene expression at the transcriptional level [24]. However, we suggest that *GHRL* RNA motif alterations might contribute to the differential level of several physiological ghrelin functions in the studied ovine–caprine population through inducing alternative transcriptional modifications. The variability in the RNA motifs has been demonstrated to generate a variety of alternatively spliced transcripts [25], or even altered protein structures [26], which have been shown to exhibit different metabolic activities on their corresponding products. Therefore, the currently observed *GHRL* mutations have potential motivation to induce a remarkable alteration in the resulting ghrelin function through producing unique RNA motifs in each observed diplotype. For instance, as *GHRL* gene powerfully increases food intake in diverse species [27], a significant role of the observed *GHRL* alterations may contribute to the food uptake status between the different species and even within the same species. In the latter case, the exclusive appearance of AB diplotype in Awassi sheep may participate in this phenomenon. Unfortunately, no reliable recording data in all Iraqi ovine and caprine breeds are available to verify this suggestion. Nonetheless, the significance of the discriminative power of the 262-bp fragment may occupy a more important role between sheep and goats. This role has a considerable impact since the feeding strategies have much more noticeable differences between ovine and caprine flocks [8]. Therefore, the absence of BB and AB diplotypes in the caprine DNA can potentially be connected to this natural phenomenon that accommodated in each species.

Our *GHRL*-based phylogenetic tree has highlighted a particular phylogenetic position for the investigated species which can be recognized easily from other related ovine–caprine species. However, this unique positioning could be attributed to the scarcity of deposited genomic sequences of the Awassi sheep, Karadi sheep, Shami goats, and Iraqi Native goats in the NCBI database. The construction of a phylogenetic tree is necessary in the presently employed *GHRL* amplicons. This may be attributed to the potential versatile role of the *GHRL* gene in the feeding strategy differences amongst these species to keep their adaptation to an ever-changing environment. In addition to feeding strategy alteration, several other ghrelin-related physiological characteristics can potentially be linked with the observed SSCP banding patterns in the *GHRL* 262-bp fragment. Therefore, a phenotypic association study is highly recommended to confirm this highly interesting distribution of SSCP banding patterns.

## Conclusions

Based on the results of the present experiments, the conclusion arises that one of the most remarkable features of the *GHRL* gene in this study is its high degree of the ovine intronic polymorphism particularly within Awassi sheep, while its caprine counterpart has exerted less polymorphic status. This locus has a remarkable ability to evolutionary differentiate between sheep and goats and between Awassi and Karadi sheep breeds too. Besides, the pattern of 262-bp fragment polymorphism may provide an indicator to understand the mechanism(s) by which *GHRL* variations may influence the gene expression pattern in both ovine and caprine species.

## Data Availability

Not applicable

## Abbreviations

acc. no.: Accession number
exp-Het: Expected heterozygosity
*GHRL*: Ghrelin
HW: Hardy–Weinberg equilibrium
NCBI: National Center of Biotechnology Information
NCBI-blastn: NCBI-blast nucleotide
obs-Het: Observed heterozygosity
PCR-SSCP: Polymerase chain reaction–single-strand conformation polymorphism
